# *In-situ* observation of ultrafast 90° domain switching under application of an electric field in (100)/(001)-oriented tetragonal epitaxial Pb(Zr_0.4_Ti_0.6_)O_3_ thin films

**DOI:** 10.1038/s41598-017-09389-6

**Published:** 2017-08-29

**Authors:** Yoshitaka Ehara, Shintaro Yasui, Takahiro Oikawa, Takahisa Shiraishi, Takao Shimizu, Hiroki Tanaka, Noriyuki Kanenko, Ronald Maran, Tomoaki Yamada, Yasuhiko Imai, Osami Sakata, Nagarajan Valanoor, Hiroshi Funakubo

**Affiliations:** 1 Department of Innovative and Engineered Material, Tokyo Institute of Technology, Yokohama, 226-8502 Japan; 2 Laboratory for Materials and Structures, Tokyo Institute of Technology, Yokohama, 226-8503 Japan; 3 Institute for Materials Research, Tohoku University, 2-1-1 Katahira, Aoba-ku, Sendai, 980-8577 Japan; 4 Materials Research Center for Element Strategy, Tokyo Institute of Technology, Yokohama, 226-8503 Japan; 5 School of Materials and Chemical Technology, Tokyo Institute of Technology, Yokohama, 226-8502 Japan; 6 School of Materials Science and Engineering, University of New South Wales, NSW 2052 Sydney, Australia; 7 Department of Materials, Physics and Energy Engineering, Nagoya University, Nagoya, 464-8603 Japan; 8 PRESTO, Japan Science and Technology Agency, 4-1-8 Honcho, Kawaguchi, Saitama, 332-0012 Japan; 9 Japan Synchrotron Radiation Research Institute (JASRI), 1-1-1 Kouto, Sayo-cho, Sayo-gun, Hyogo, 679-5198 Japan; 10 Synchrotron X-ray Station at SPring-8 and Synchrotron X-ray Group, National Institute for Materials Science (NIMS), 1-1-1 Kouto, Sayo, Hyogo, 679-5148 Japan

## Abstract

Ferroelastic domain switching significantly affects piezoelectric properties in ferroelectric materials. The ferroelastic domain switching and the lattice deformation of both *a*-domains and *c*-domains under an applied electric field were investigated using *in-situ* synchrotron X-ray diffraction in conjunction with a high-speed pulse generator set up for epitaxial (100)/(001)-oriented tetragonal Pb(Zr_0.4_Ti_0.6_)O_3_ (PZT) films grown on (100)_*c*_SrRuO_3_//(100)KTaO_3_ substrates. The *004* peak (*c*-domain) position shifts to a lower 2*θ* angle, which demonstrates the elongation of the *c*-axis lattice parameter of the *c*-domain under an applied electric field. In contrast, the *400* peak (*a*-domain) shifts in the opposite direction (higher angle), thus indicating a decrease in the *a*-axis lattice parameter of the *a*-domain. 90° domain switching from (100) to (001) orientations (from *a*-domain to *c*-domain) was observed by a change in the intensities of the *400* and *004* diffraction peaks by applying a high-speed pulsed electric field 200 ns in width. This change also accompanied a tilt in the angles of each domain from the substrate surface normal direction. This behaviour proved that the 90° domain switched within 40 ns under a high-speed pulsed electric field. Direct observation of such high-speed switching opens the way to design piezo-MEMS devices for high-frequency operation.

## Introduction

Ferroelectric materials have been applied to many electronic devices, including sensors and actuators. Because of their superior piezoelectric and ferroelectric properties, Pb(Zr_1−*x*_Ti_*x*_)O_3_ thin films have been extensively studied for microelectromechanical system [MEMS] applications^1^. It has been pointed out that the electromechanical response of Pb(Zr_1−*x*_Ti_*x*_)O_3_ is composed of not only lattice elongation (so-called intrinsic contribution of piezoresponse) but also extrinsic contributions, which include the motion of the domain walls, separating ferroelastic domains (non-180° domain motion) under an applied electric field, for example, switching from (100)- to (001)-orientated domains (90° domain switching) in tetragonal Pb(Zr_1−*x*_Ti_*x*_)O_3_ films^2^. In particular, the extrinsic contributions are responsible for more than 50% of the total piezoelectric response, as well as the dielectric response^3^. Therefore, non-180° ferroelastic domain switching induced by the application of an electric field is a significant feature and is required for the design of high-performance piezoelectric devises for MEMS applications. Much efforts have been devoted to clarify the dynamics of the non-180° ferroelastic domain in epitaxial Pb(Zr_1−*x*_Ti_*x*_)O_3_ films^4–9^. The change in the non-180° ferroelastic domain structure with the application of an electric field has been investigated using various techniques, such as transmission electron microscopy (TEM)^6, 9^, piezoresponse force microscopy (PFM)^4, 7, 8^, and X-ray diffraction (XRD)^5^ for polydomain (100)/(001)-oriented epitaxial tetragonal Pb(Zr_1−*x*_Ti_*x*_ **)**O_3_ films. The domain structures strongly depend on the film thickness and substrate^10–12^, and the Zr/(Zr + Ti) ratio in the films^13^, as well as the poling treatment.

The dynamics of the crystal lattice while applying an electric field in Pb(Zr_1−*x*_Ti_*x*_)O_3_ films have been measured by *in-situ* XRD measurement^14–17^. Lattice elongation and 180° domain wall motion were observed at sub-nanosecond timescales for (001)-oriented Pb(Zr_1−*x*_Ti_*x*_)O_3_ thin films^14, 15^, and it has been reported that non-180° ferroelastic domains can be switched by application of an electric field on the order of hundreds of nanoseconds^16, 17^ in ferroelectric films. However, it has not been clarified yet how fast does the 90° domain switching proceed - in particular, the relationship between the lattice elongation and geometrical domain structure deformation in tetragonal Pb(Zr_1−*x*_Ti_*x*_)O_3_ thin films has not been determined from a response speed point of view.

In the present study, we investigated the change in lattice parameters and domain structure for 600-nm thick (100)/(001)-oriented epitaxial tetragonal Pb(Zr_0.4_Ti_0.6_)O_3_ films, which were subjected to an applied rectangular electric field pulses of 200-ns and rest time of 800 ns. The average lattice parameters and domain structure of the films under this electric field was then determined using a time-resolved *in-situ* synchrotron X-ray diffraction system in conjunction with a high-speed pulse generator. We show that in contrast to conventionally accepted scenario of sluggish nature of ferroelastic domain switching as pointed out in ceramics^18^, simultaneous lattice deformation (*c*-axis and *a*-axis) and ferroelastic domain switching (*c*-domain volume fraction and geometrical twin 90° domain structure) occurs on the order of sub-microseconds.

## Results and Discussion

**Figure 1:**
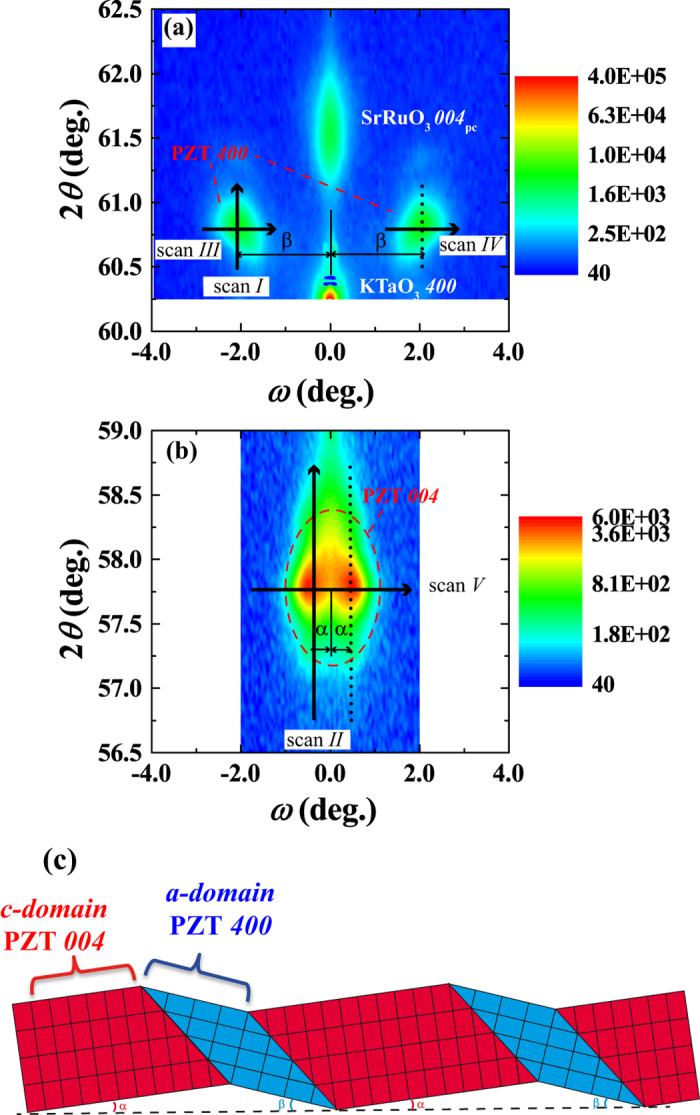
HRXRD 2*θ*-*ω* mappings near (**a**) PZT *400* and (**b**) PZT *004* for (100)/(001)-oriented epitaxial tetragonal Pb(Zr_0.4_Ti_0.6_)O_3_ films. (**c**) Schematic representation of the domain structure.

High-resolution X-ray diffraction (HRXRD) 2*θ*- *ω* mappings around the PZT *400* and *004* diffraction peaks of (100)/(001)-oriented epitaxial PZT films grown on (100)_*c*_SrRuO_3_//(100)KTaO_3_ substrates are shown in Fig. 1(a) and (b), respectively. Split PZT *400* diffractions as twin peaks (*a*-domains) are observed in Fig. 1(a), together with SrRuO_3_ *400*_c_ and KTaO_3_ *400* diffraction peaks, while split PZT *004* diffractions as twin peaks (*c*-domains) are observed in Fig. 1(b). This tilted domain structure has been examined in a number of reports using cross-sectional TEM or X-ray rocking curve analysis^10–13, 19–24^. The twin peaks split in the rocking curve direction (*ω*- axis), the horizontal axis in Fig. 1(a) and (b), are observed because of the tetragonality of the film structure^10, 12^. The tilting angles of *c*-domains (*β* angle) and *a*-domains (*α* angle) can be interpreted by considering that the films are required to be attached to a flat surface substrate, as illustrated in Fig. 1 (c). Angles of *α* = 0.41° and *β* = 1.96° were obtained from the 2*θ*- *ω* mappings shown in Fig. 1(a) and (b), respectively. It must be mentioned that the disoriented angles from the substrate surface have been ascertained to depend on the volume fraction of the *c*-domain (*V*_c_)^22^ and the tetragonality (*c*/*a* ratio), as described in supplementary materials^12^.

**Figure 2:**
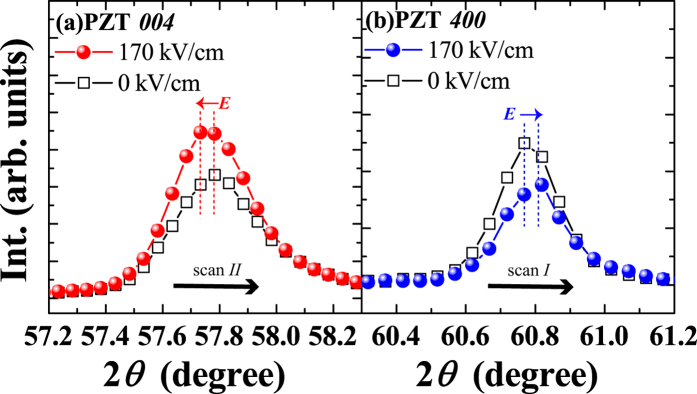
HRXRD 2*θ* scans of (**a**) PZT *004* [scan *II* in Fig. 1(b)] and (**b**) PZT *400* [scan *I* in Fig. 1(a)] measured under a 200-ns pulsed electric field with amplitudes of 0 kV/cm (open squares) and 170 kV/cm (filled circles) for the same Pb(Zr_0.4_Ti_0.6_)O_3_ films shown in Fig. 1.

Figure 2(a) and (b) show HRXRD 2*θ*-*ω* scans for PZT *004* [scan *II* in Fig. 1(b)] and PZT *400* [scan *I* in Fig. 1(a)] measured by the application of a 200-ns pulsed electric field with an amplitude of 170 kV/cm and zero field. The PZT *004* peak shifted to a lower 2*θ* angle, which demonstrates the elongation of the surface normal *c*-axis lattice parameter of the *c*-domain, with an applied electric field. On the other hand, the PZT *400* peaks shifted in the opposite direction (higher 2*θ* angle), as shown in Fig. 2(b), indicating a decrease in the surface normal *a*-axis lattice parameter of the *a*-domain under an applied electric field. This elongation of the surface normal *c*-axis lattice parameter of the *c*-domain and decrease in the surface normal *a*-axis lattice parameter of the *a*-domain occurs simultaneously. Moreover, a clear difference in the intensities of both PZT *004* and PZT *400* diffraction peaks between 0 V (before applying an electric field) and 170 kV/cm (under applying an electric field) is found. This result is direct evidence of 90° domain switching from (100) to (001) orientation, as is the increase in *V*_c_ during a 200-ns-wide pulsed electric field. The application of an electric field forces ferroelectric/ferroelastic domains to move towards the direction parallel to the electric field to minimise the total energy in the film. It must be noted that the decrease in the lattice spacing along the *a*-axis of *a*-domain that is perpendicular to the polarization direction can be understood by the shear deformation owing to none-zero piezoelectric tensor *d*_15_^25^. The appreciable *d*_15_ response in the *a*-domain may be also associated with the change of domain fraction by the electric field. Full understanding of the geometrical correlation between *a*- and *c*-domains under the electric field will be reported in the future.

**Figure 3:**
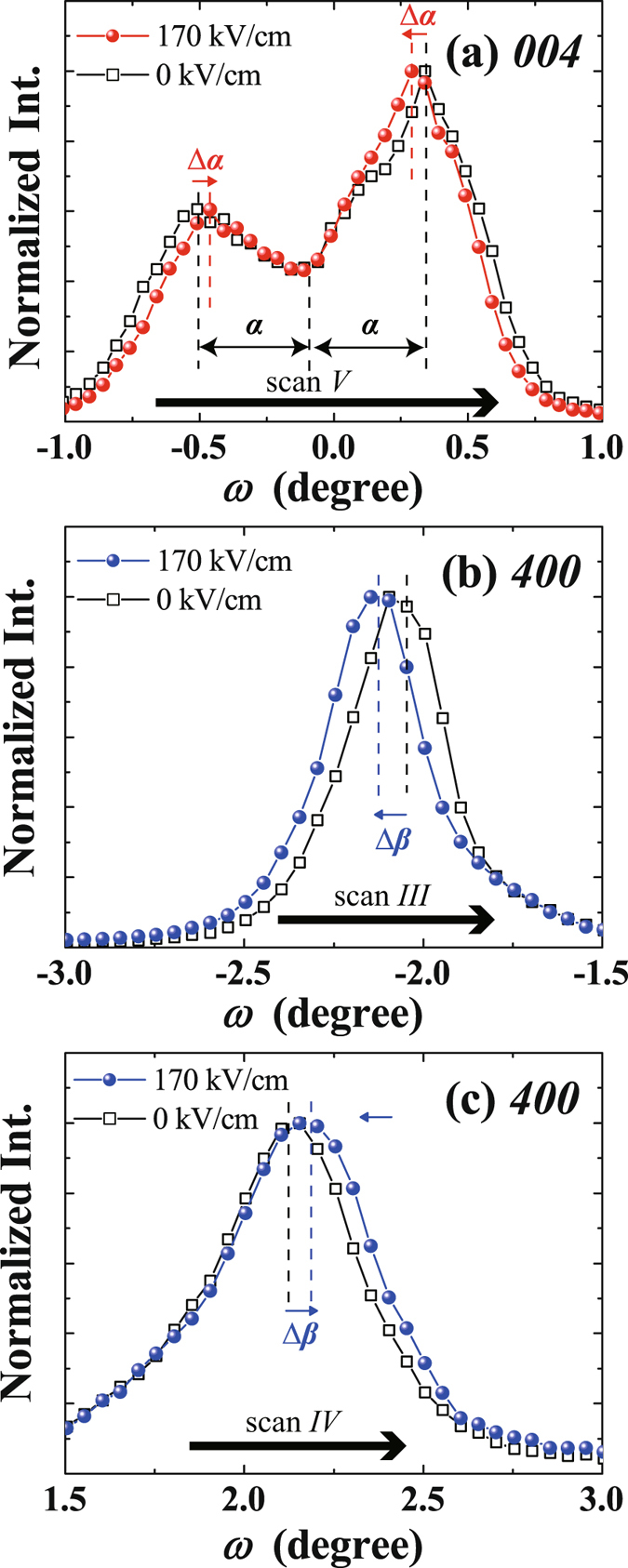
Rocking curves (*ω* scans) of (**a**) *004* PZT under 0 kV/cm (open squares) and 170 kV/cm (filled circles) in scan *V* in Fig. 1(b). (**b**,**c**) Rocking curve of *400* PZT under 0 kV/cm (open squares) and 170 kV/cm (filled circles) of image in (**b**) scan *III* and (**c**) scan *IV* in Fig. 1(a), respectively.

Figure 3 shows the change in the rocking curve scans (*ω* scans) for the *c*-domain [scan *V* in Fig. 1(b)] and *a*-domain [scans *III* and *IV* in Fig. 1(a)] under an applied 200-ns-wide pulsed electric field. The tilting angle of the PZT *004* twin peaks decreased, as shown in the Fig. 3(a). This motion indicates decreasing *α* angles of the *c*-domains (*Δ* *α*), which is accompanied by an increase in *V*_c_ under the applied electric field, in agreement with Fig. 2. On the other hand, as shown in Fig. 3(b) and (c), the PZT *400* peaks were shifted to lower and higher angles, respectively, which indicate an increase in the *β* angle of the *a*-domains (*Δβ*) under the application of an electric field. Thus the time-resolved *in-situ* XRD measurements show direct evidence of a dynamic increase of the *V*_c_ and lattice parameters as well as changing tilting angle of each domain with the application of an electric field.

**Figure 4:**
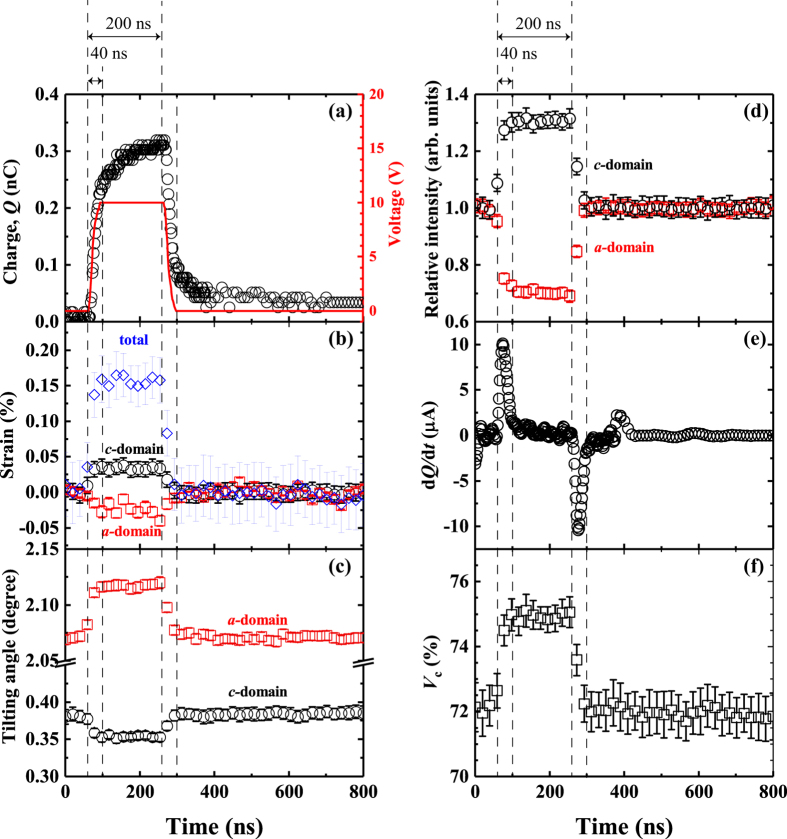
(**a**) Capacitance and (**e**) calculated differential capacitance by time as a function of time during application of a 200-ns pulsed electric field with a magnitude of 170 kV/cm. The solid line in panel (**a**) indicates applied pulse voltage measured by reference capacitor. (**b**) Strain, (**c**) tilting angle, (**d**) intensity, and (f)*V*_*c*_ of PZT *400* (circles) and PZT *004* (squares) peaks as a function of time during application of a 200-ns pulsed electric field with a magnitude of 170 kV/cm. Iintensities are integrated one obtained by peak fitting. Open circles and squares are calculated by the *004* diffraction peak from the *c*-domain and the *400* diffraction peak from the *a*-domain, respectively. Total strain including extrinsic contribution (open diamond) was also plotted in panel (b).

We now discuss the time response of the changes in the lattice parameters, *V*_*c*_, and the tilting angle of the domains with respect to an electric field. Figure 4 shows the electric charge (Fig. 4(a)), the lattice strains (Fig. 4(b)), the tilting angles (Fig. 4(c)), and the peak intensity of each domain (Fig. 4(d)) as a function of time. The electrical charges were plotted every 2 ns, while others were every 20 ns to obtain sufficient intensity for analysis. These data were obtained by fitting with Gaussian functions except for Fig. 4(a). An imperfect rectangular shape of the charge with respect to an applied electric pulse is shown in Fig. 4(a) when a 200-ns-wide pulsed voltage with amplitude of 170 kV/cm was applied to the PZT films. To further visualise this in detail, the time derivative for the charge, which is the current flowing through the electric circuit, is shown in Fig. 4(e). Two peaks correspond to the current associated with applying and removing an electric field. The transition duration, which is defined as the time over which a current with an absolute value larger than the noise level of 0.002 mA flows, is approximately 40 ns for both application and removal. This duration time includes both rise time, determined by the limitation of the current pulse generator, and transient time, determined by impedance of the electric circuit. Conversely, Fig. 4(b)-(d) show that the elastic deformation, tilting motion, and ferroelastic domain switching seem to have been completed within 40 ns because each spectrum has only one single data point during the change in electric field that corresponds to these transition states. As previous studies reported^13^, the tilting angle is closely related to the volume fractions of each domain and tetragonality of the crystal lattice. This change in tilting angle indicates the change in the volume fraction of *c*-domain. Unfortunately, the volume fraction of domains cannot be estimated by the time dependent intensities of *400* and *004* peaks due to complexity of the domain structures. But the increase and decrease in integrated intensities of *004* and *400* peaks, respectively, strongly support the domain switching from *a*-domain to *c*-domain. When the field was removed, tilt angles and intensities return back to their original positions at zero field at less than 40 ns as well. These data were acquired during the repetitive application of electric pulses of 800-ns duration confirming that the observed changes in tilt angles and intensities and hence a ferroelastic domain wall motions is perfectly repeatable. More importantly all these motions (charge, lattice strain, domain switching, and change in tilting angle) occur simultaneously without delay when the electric field across the PZT film changed. This means that the 90° domain switching from (100) to (001) can be exploited to enhance the piezoresponse response even on the order of several tens of nanoseconds. It should be noted that the response time in this study is less than that of previously reported data, which is on the order of hundreds of nanoseconds^16, 17^. Moreover, the non-180° ferroelastic domain motion for thin films does not suffer from frequency dispersion as seen in bulk ceramics^18^, where it becomes inactive for short such ns pulse time. One possible explanation could be that the electric fields applied to the film are one order higher than those applied to the ceramics, which may have enabled us to achieve high-speed non-180° domain switching. Additionally non-180° domain switching by an electric field can be also influenced by strains of the grain that are accumulated in polycrystalline ceramic bodies. These are changed by grain size, grain shape, and grain-to-grain disorientation in neighbour grains^26^.

Finally, it is worth noting to compare the strain evaluated from the present *in-situ* XRD and strain-electric-field measurement (see Fig. S3) by applying unipolar electric field with 5 Hz. The figure shows that the almost linear response of strain with respect to electric field, indicating piezoelectric coefficient (***d***_33obs._) of around 100 pC/N. The volume fraction change in *c*-domain calculated from tilting angle and total strain as a function of time during *in-situ* XRD measurements are plotted in Fig. 4(f) and (b), respectively. The piezoelectric coefficient (***d***_33XRD_) obtained from the XRD measurement is around 86 pC/N, showing reasonable agreement with the value obtained by strain - electric field measurements. This consistency between them again confirms that the ferroelastic domain switching here does not suffer from strong frequency dispersion previously observed for 90° domain switching^18^.

Note that the intrinsic component, which indicates the lattice deformation of *c*-domain by electric field, is only 17 pC/N, of which contribution for total ***d***_33XRD_ is around 20%. That is domain switching from *a*-domain to *c*-domain dominates the total piezoelectric response. Our observation of fast 90° domain switching opens up exciting new possibilities for ultrafast electromechanical switches and sensors that including piezoelectric transistor applications that rely on 90° domain wall motion^27^.

## Conclusion

In summary, we used time-resolved synchrotron XRD in conjunction with a high-speed pulse generator to demonstrate the average lattice and domain motions as well as the tilting angle of the domains. The 90° domain can be switched by a 200-ns-wide electric field. The change in tilt angles of *Δα*  = 0.05° in the *a*-domain and *Δβ* = −0.03° in the *c*-domains were observed under an applied electric field, commensurate with a change in *V*_c_ and tetragonality (*c/a*). Moreover, reversible 90° occurs within 40 ns, a limit set by our present electrical set up. This opens up exciting new possibilities for ultrafast electromechanical switches and sensors that rely on 90° domain wall motion.

## Methods

Pb(Zr_0.4_Ti_0.6_)O_3_ [PZT] films, 600 nm in thickness, grown on (100)_c_SrRuO_3_-coated (100) KTaO_3_ substrates by pulsed-metal organic chemical vapour deposition (MOCVD) were used for measurements. Details of the sample preparation are given elsewhere^28^. SrRuO_3_, which was indexed by a pseudo-cubic notation like *hkl*_pc_ here, was grown by a RF-magnetron sputtering method reported in our previous paper^29^. For applying an electric field to this film, Pt top electrodes 100 μm in diameter were fabricated on the PZT films by electron-beam evaporation.

To determine the structural characterisations of the PZT films before applying an electric field, high-resolution X-ray diffraction (HRXRD) and 2*θ*- *ω* mapping were performed with a 12.4-keV X-ray beam at the National Institute for Materials Science (NIMS) beam line, BL15XU, of SPring-8, Japan.

*In-situ* observation of the change in lattice parameters and domain structure with an applied electric field was carried out at the BL13XU at SPring-8 for Pt/PZT/SrRuO_3_ capacitors. To acquire the electric field-induced changes in diffraction data, an incident beam with photon energy of 12.4 keV was focused down to a few micrometres in width and height using a two-dimensional focusing refractive lens and irradiated on the Pt top electrode with the help of a fluorescent X-ray detector. We applied pulse voltages with a magnitude of 10 V and monitored the changes in *400* and *004* diffraction peaks. A positive 10-V square pulsed voltage 200 ns in width was applied to the capacitor to obtain sufficient poling treatment prior to measurement. The 200-ns square pulsed electric field was applied using a pulse generator (Agilent, 8114 A) and FCE-HS3 (Toyo, 6321). Time-resolved X-ray diffraction was measured using a high-speed avalanche photodiode detector (APD). The pulse height generated by a time-to-amplitude converter (ORTEC 567), for which X-ray pulse measured by the APD detector was used as a start signal and pulse signal synchronized to the electric pulse applied to the PZT films was used as a stop signal, is canalized by a multi-channel analyser. This technique cannot detect more than one photon for one period, so that properly attenuated X-rays were irradiated. No remarkable fatigue was confirmed during measurements (see Fig. S4). Detailed description of the time-resolved synchrotron X-ray diffraction measurement is given elsewhere^30^.

## Electronic supplementary material

Supplementary Information 
