# Supplementary material for: In-situ observation of ultrafast 90° domain switching under application of an electric field in (100)/(001)-oriented tetragonal epitaxial Pb(Zr0.4Ti0.6)O3 thin films
Source: Sci Rep. 2017 Aug 29;7:9641. doi: 10.1038/s41598-017-09389-6 (PMC5575037; doi:10.1038/s41598-017-09389-6)
Supplement: Supplementary file 1 — Supplementary Information [file 41598_2017_9389_MOESM1_ESM.pdf]

In-situ observation of ultrafast 90° domain switching under application of an electric field in (100)/(001)-oriented tetragonal epitaxial $Pb(Zr_{0.4}Ti_{0.6})O_3$ thin films

Yoshitaka Ehara[1], Shintaro Yasui[2], Takahiro Oikawa[1], Takahisa Shiraishi[1,3], Takao Shimizu[4,5], Hiroki Tanaka[1], Noriyuki Kanenko[1], Ronald Maran[6], Tomoaki Yamada[7,8], Yasuhiko Imai[9], Osami Sakata[1,10], Nagarajan Valanoor[6], and Hiroshi Funakubo[*1,4,5]

1. Department of Innovative and Engineered Material, Tokyo Institute of Technology, Yokohama 226-8502, Japan
2. Laboratory for Materials and Structures, Tokyo Institute of Technology, Yokohama 226-8503, Japan
3. Institute for Materials Research, Tohoku University, 2-1-1 Katahira, Aoba-ku, Sendai 980-8577, Japan
4. Materials Research Center for Element Strategy, Tokyo Institute of Technology, Yokohama, 226-8503, Japan
5. School of Materials and Chemical Technology, Tokyo Institute of Technology, Yokohama 226-8502, Japan

6. School of Materials Science and Engineering, University of New South Wales, NSW 2052 Sydney Australia

7. Department of Materials, Physics and Energy Engineering, Nagoya University, Nagoya 464-8603, Japan

8. PRESTO, Japan Science and Technology Agency, 4-1-8 Honcho, Kawaguchi, Saitama 332-0012, Japan

9. Japan Synchrotron Radiation Research Institute (JASRI), 1-1-1 Kouto, Sayo-cho, Sayo-gun, Hyogo 679-5198, Japan

10. Synchrotron X-ray Station at SPring-8 and Synchrotron X-ray Group, National Institute for Materials Science (NIMS), 1-1-1 Kouto, Sayo, Hyogo, 679-5148, Japan

*Author to whom correspondence should be addressed. Electric mail:

funakubo.h.aa@m.titech.ac.jp

1. Domain structure of PZT film

Figure S1 shows the plan view measurements for PZT film on $KTaO_3$ substrate used in this study. As shown in Fig. S1(a), the *c*-domain tilt with respect to substrate surface. Associated with this, additional *a*-domain variants tilt to originally existing *a*-domain variant, as marked by solid circles in Fig. S1(b).

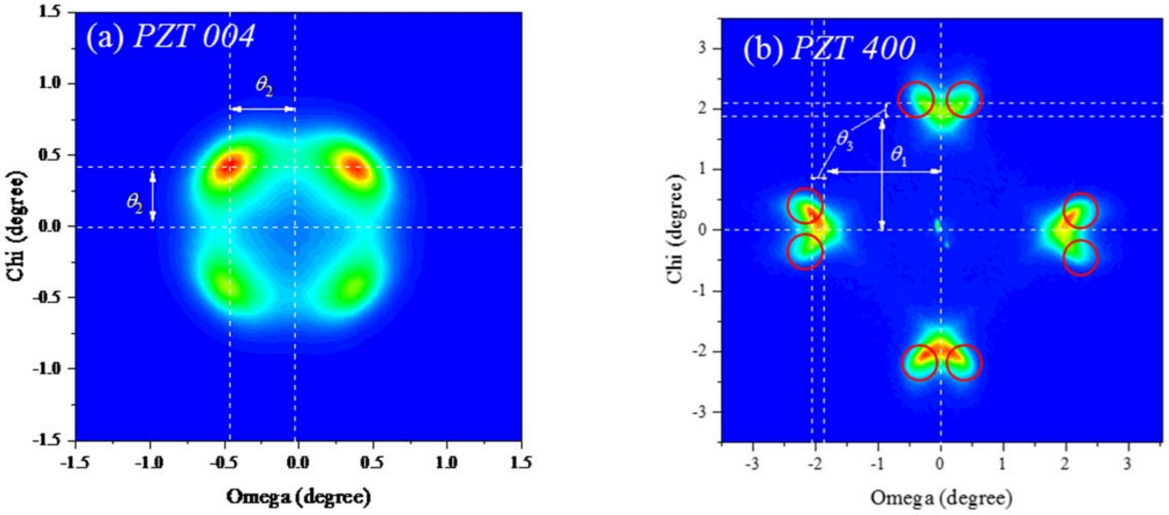

Figure S1. XRD plan view measurements for PZT (a) *004* and (b) *400* reflections. The regions circled by red circles correspond to additional variants.

The detailed analysis are given in previous studies. In this domain structure, it is reported that the tilting angles (θ_1, θ_2, and θ_3) are connected with *c*-domain volume fraction (V_c) by the following equation;[1-3]

$$V_c = \frac{\theta_1}{\theta_1+\theta_2+\theta_3} \quad \text{(S1)}$$

The time-dependent rocking curve measurements (scan *III*, *IV*, and *V*) are performed without inclination (at chi = 0 in Fig. S1) so that the obtained α and β do not correspond to θ_1, θ_2, and θ_3. To avoid this complexity, the rocking curves were measured with widely opened horizontal slit, which enables to collect for wide chi range (low resolution for chi direction). In addition, it is known that θ_2 is equal to θ_3.[1] By taking this into account, we can assume the following relations,

$$\alpha = \theta_2 \,, \tag{S2}$$

$$\beta = \theta_1 + \frac{\theta_3}{2} = \theta_1 + \frac{\theta_3}{2} \,. \tag{S3}$$

Based on these assumption, V_c can be represent with obtained α and β by the following equation,

$$V_c = \frac{2\beta - \alpha}{3\alpha + 2\beta} \,. \tag{S4}$$

Figure S2 shows plan view measurement for PZT *400* reflections recorded with focused X-ray beam by 2 dimensional lens.

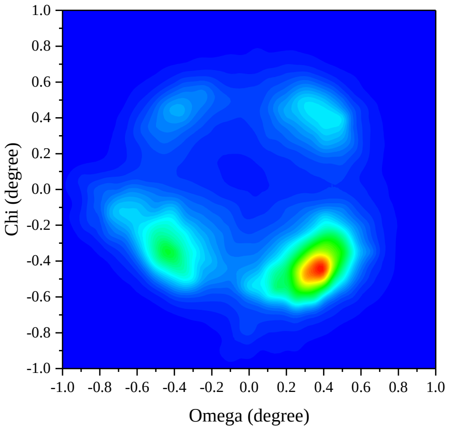

Figure S2. XRD plan view measurement for PZT *004* reflections with focused X-ray beam shaped with two dimensional lens.

An asymmetric distribution of all 4 variants can be observed. This asymmetric fraction of variants can cause the asymmetric peak shape observed in Fig. 3(a), however the fact we observe all 4 variants enables us to evaluate the tilting angle.

2. Piezoelectric and ferroelectric characterization of the PZT film.

Figure S3 shows the results of ferroelectric and piezoelectric characterization by ferroelectric tester combination with atomic force microscopy. The measurement of electric displacement – electric field (***D***-***E***) hysteresis and displacement – electric voltage measurement are performed by 1 kHz bipolar triangle field and 5 Hz unipolar triangle field.

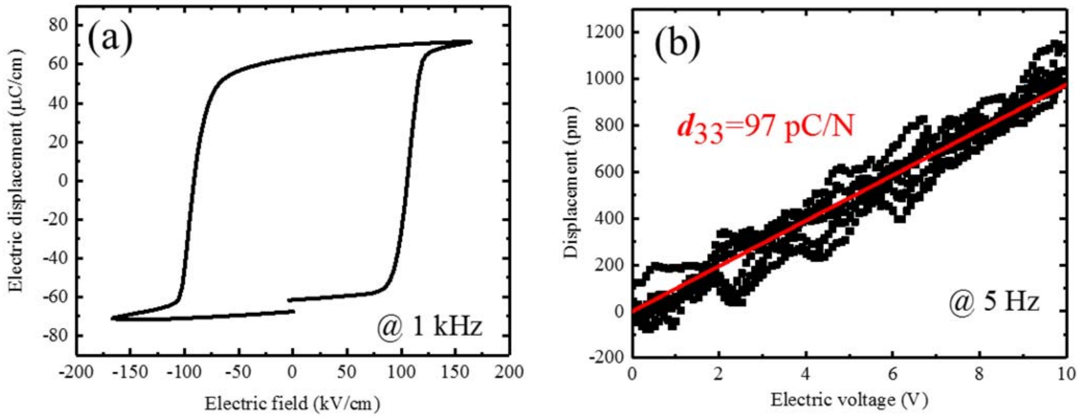

Figure S3. (a) ***D-E*** hysteresis curve and displacement as a function of electric voltage measured by ferroelectric tester. The displacement was measured by atomic force microscopy set up.

The saturated polarization of around 60 μC/cm^2 estimated by Fig. S3(a), in agreement with V_c of around 70% (the estimated spontaneous polarization of fully *c*-axis-oriented PZT with

Zr/(Zr+Ti) = 0.4 is ~ 80 μC/cm^2).[2,4] The piezoelectric coefficient $\boldsymbol{d}_{33}$ of 97 pC/N is obtained by linear fitting for displacement-electric voltage curve.

3. Fatigue characterization of PZT film during *in-situ* measurements.

Figure S4 shows the electric charge-time curves measured before *in-situ* measurement and after all time-dependent scans. No obvious different between them indicates that no fatigue failure occurs during *in-situ* XRD measurements.

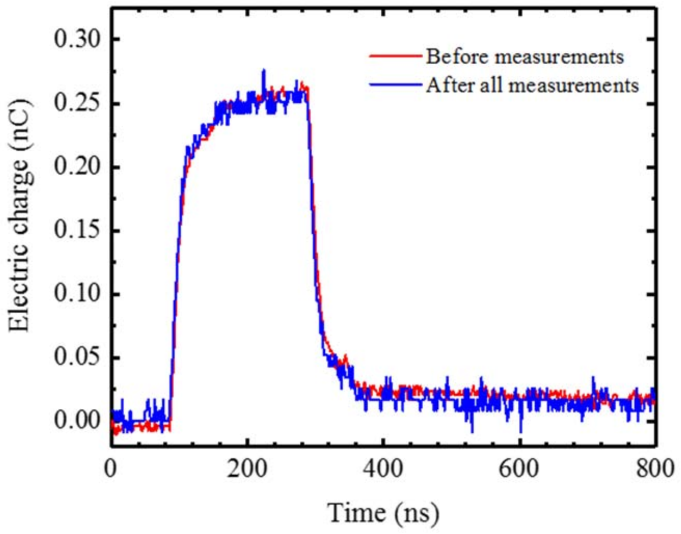

Figure S4. Time dependent electric charge curve measured before (red) and after (blue) *in-situ* XRD measurements.
